# Structures, Biosynthesis, and Physiological Functions of (1,3;1,4)-β-d-Glucans

**DOI:** 10.3390/cells10030510

**Published:** 2021-02-27

**Authors:** Shu-Chieh Chang, Rebecka Karmakar Saldivar, Pi-Hui Liang, Yves S. Y. Hsieh

**Affiliations:** 1 Division of Glycoscience, Department of Chemistry, School of Engineering Sciences in Chemistry, Biotechnology and Health, Royal Institute of Technology (KTH), AlbaNova University Centre, SE-106 91 Stockholm, Sweden; scch@kth.se (S.-C.C.); karmak@kth.se (R.K.S.); 2 School of Pharmacy, College of Pharmacy, Taipei Medical University, Taipei 110, Taiwan; 3 School of Pharmacy, College of Medicine, National Taiwan University, Taipei 100, Taiwan; phliang@ntu.edu.tw; 4 Genomics Research Center, Academia Sinica, Taipei 115, Taiwan

**Keywords:** cell wall, polysaccharide, (1,3;1,4)-β-d-glucan, mixed-linkage glucan

## Abstract

(1,3;1,4)-β-d-Glucans, also named as mixed-linkage glucans, are unbranched non-cellulosic polysaccharides containing both (1,3)- and (1,4)-β-linkages. The linkage ratio varies depending upon species origin and has a significant impact on the physicochemical properties of the (1,3;1,4)-β-d-glucans. (1,3;1,4)-β-d-Glucans were thought to be unique in the grasses family (Poaceae); however, evidence has shown that (1,3;1,4)-β-d-glucans are also synthesized in other taxa, including horsetail fern *Equisetum*, algae, lichens, and fungi, and more recently, bacteria. The enzyme involved in (1,3;1,4)-β-d-glucan biosynthesis has been well studied in grasses and cereal. However, how this enzyme is able to assemble the two different linkages remains a matter of debate. Additionally, the presence of (1,3;1,4)-β-d-glucan across the species evolutionarily distant from Poaceae but absence in some evolutionarily closely related species suggest that the synthesis is either highly conserved or has arisen twice as a result of convergent evolution. Here, we compare the structure of (1,3;1,4)-β-d-glucans present across various taxonomic groups and provide up-to-date information on how (1,3;1,4)-β-d-glucans are synthesized and their functions.

## 1. Introduction

β-Glucans have been found to be highly abundant in plants, algae, fungi, and bacteria as one of the fundamental fibers in the cell walls. The polysaccharides are composed of D-glucopyranosyl units (Glc*p*) as building blocks. Depending on the glycosidic bonds between the glucose monomers, β-glucans can be classified into two sub-groups, cereal and non-cereal β-glucans. For example, the yeast and fungi β-glucans contain mainly 1,3 and branching 1,6 linkages, whereas cereal β-glucans have linear glucan chain composed of 1,3 and 1,4 glycosidic linkages. (1,3;1,4)-β-d-Glucans, or mixed-linkage glucans (MLGs), have been found rich in rice, wheat, cereal grains and oats, serving as important dietary fibers for our daily consumption, and also impact our metabolic activities by decreasing cholesterol and blood glucose [1].

**Figure 1:**
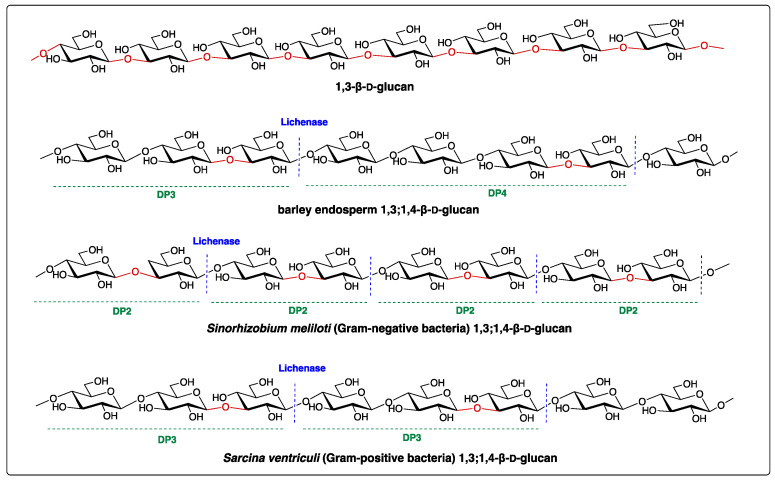
Structures of 1,3-β-d-glucan and (1,3;1,4)-β-d-glucans. Lichenase is an *endo*-hydrolase that hydrolyses the (1,3;1,4)-β-d-glucans into smaller oligomers, such as degree of polymerization 2 (DP2), DP3 and DP4 shown above. The blue dash lines represent the enzymatic cleavage sites. Oligosaccharide profiles in DPs vary depending on the taxonomic origin of (1,3;1,4)-β-d-glucans.

Structurally, the (1,3;1,4)-β-d-glucan is a linear polymer, primarily containing (1,4)-linked β-d-glucose residues interspersed with a single (1,3)-β-linkage. The ratio of (1,3)- to (1,4)-β-linkages is a good indicator to the fine structure of (1,3;1,4)-β-d-glucan and determines its solubility and physiochemical properties [2]. The polysaccharide can be broken down by the specific polysaccharide hydrolase, the lichenase (EC 3.2.1.73), which cleaves at a (1,4)-β-d-glucopyranosyl linkage immediately adjacent to a (1,3)-β-d-glucopyranosyl residue, yielding oligosaccharides with distinctive structures such as the cellotriose Glc*p*-β-1,4-Glc*p*-β-1,3-Glc*p* (G4G4G3G_R_, with degree of polymerization of three, namely DP3) and cellotetraose Glc*p*-β-1,4-Glc*p*-β-1,4-Glc*p*-β-1,3-Glc*p* (G4G4G4G3G_R_, DP4) (Figure 1) [3]. Since this discovery, the lichenase has been essential in the structural profiling of (1,3;1,4)-β-d-glucans. For example, the enzymatic digestion of (1,3;1,4)-β-d-glucans of cereal and grasses (family Poaceae), such as barley, results in a higher proportion of DP3 compared to DP4, with a ratio of 1.5–4.5 [4,5,6], whereas the (1,3;1,4)-β-d-glucans of horsetail (*Equisetum spp.*) of a monilophyte family Equisetaceae *sensu stricto* releases predominantly DP4 after lichenase digestion (Figure 1 and Table 1) [3,7,8]. The structure of (1,3;1,4)-β-d-glucans of the brown algae *Ectocarpus sp.* has been recently reported to have only DP3 repeating units (Table 1) [9]. In contrast, Glc*p*-β-1,3-Glc*p* (DP2) and DP3 are the most abundant oligomers in (1,3;1,4)-β-d-glucans from *Sinorhizobium meliloti* and *Sarcina ventriculi*, respectively [3,10]. Structural surveys reveal (1,3;1,4)-β-d-glucans display heterogeneous structures between different taxonomic groups (Table 1), which suggests differential functions in the cell walls of the different organisms.

(1,3;1,4)-β-d-Glucans have been widely accepted as functional and bioactive ingredients in dietary fibers [14]. The polysaccharides are known to enhance viscosity of the solution and form a matrix-like gel under suitable conditions [14,15,16]. Hence, (1,3;1,4)-β-d-glucans have been utilized in industry as thickening agents for gravies, ice cream formulations, etc. [17], or as fat mimetics in low-calorie food [18]. Additionally, (1,3;1,4)-β-d-glucans extracted from human dietary fiber have been identified that can lower serum cholesterol, reduce the risk of type II diabetes and obesity, and provide other healthful benefits [19,20,21,22], sometimes they can also function as a source of metabolizable energy [23]. It is also observed that some bacteria contain MLG utilization loci (MLGULs), encoding genes that allowing the bacteria to utilize MLGs as energy sources. Therefore, the MLGULs can serve as genetic markers for (1,3;1,4)-β-d-glucan catabolism in commensal gut bacteria [24].

## 2. Variation of (1,3;1,4)-β-d-Glucans in Different Taxa

### 2.1. Viridiplantae

(1,3;1,4)-β-d-Glucans have been found in the cell walls of the grasses and cereal family, also known as Poaceae, which consist of commercially important cereals. During the plant growth and development, the amount of (1,3;1,4)-β-d-glucans in the wall is found to increase proportionally to the cell elongation rate, reaching its maximum during the most rapid phase of cell growth, and is completely hydrolyzed when growth ceases. Not only functioning as structural elements, the (1,3;1,4)-β-d-glucans are present in high abundance in the walls of aleurone layer surrounding the barley, rye, and oats starchy endosperms. These (1,3;1,4)-β-d-glucans are hydrolyzed by specific enzymes, the (1,3;1,4)-β-d-glucanases, when germination occurs, allowing mobilization of endosperm cell walls and also providing an extra carbon source that facilitates the germination process [25,26].

By enzymatic profiling and linkage analysis, the ratio of (1,4)- to (1,3)-β-linkages is defined within the range of 2.2–2.6:1 in (1,3;1,4)-β-d-glucans of cereals, and this translates to a ratio of DP3:DP4 units ranges from approximately 1.5:1 to 4.5:1, with the occasional occurrence of longer oligosaccharide units of up to DP > 13 [6,27,28]. So far, similar oligomeric distributions have been found within related species, which indicates that the (1,3)- and (1,4)-β-linkages are not randomly generated. On the other hand, whether the differences in ordered combinations of DP3 and DP4 constitutive blocks is species-dependent is yet to be solved. Among vascular plants, *Equisetum* species, also known as horsetails, are reported to have (1,3;1,4)-β-d-glucans in their cell walls with significant lower DP3:DP4 ratio (0.05–0.1:1). Higher abundance of the DP4 repeat units may facilitate stronger interaction with the cellulosic microfibrils [3,7].

Chlorophyte green algae *Ulva lactuca* and the Bryophyte liverwort *Lophocolea bidentata* are reported to have (1,3;1,4)-β-d-glucan-like polysaccharides, but unlike the homopolymeric (1,3;1,4)-β-d-glucans in barley, their glucans tend to include xylose and arabinose, with a higher degree of polymerization in the latter [29]. (1,3;1,4)-β-d-arabinoglucan is a recently discovered polysaccharide in the walls of moss *Physcomitrella patens* [30]. It is a linear glucan-related polysaccharide with a structure similar to the (1,3;1,4)-β-d-glucan, except the three-linked Glc*p* is substituted with three-linked Ara*f* residues. These studies showed that the non-vascular plants could also produce structurally related glucans. The function of (1,3;1,4)-β-d-arabinoglucan remains for further investigation.

(1,3;1,4)-β-d-Glucans are found in the walls of red algae and brown algae, and both are evolutionarily more distant to land plants. A sulfated (1,3;1,4)-β-d-glucan has been isolated from the red algae *Kappaphycus alvarezii.* This sulfated glucan has only 180 residues, with 64% sulfation on the (1,4)-β-linkages only and the remaining (1,4)-β-linkages were sparse and unlikely to be in long sequences after one and another. The sulfated (1,4)-β-linkages likely link to the fibrillar cell wall polymers by their incompatible extraction behavior. It is also speculated that the sulfation helps control construction and positioning of cellulose [31].

In recent years, the presence of (1,3;1,4)-β-d-glucans in algae species is further supported by their initial discovery in brown algae. Remarkably, a study by Salmeán et al. on brown algae, including six clades of main orders and contains 34 species in total, showed (1,3;1,4)-β-d-glucans were present in all as detected by antibody-based glycan array analysis. Even more astonishing is that, unlike the (1,3;1,4)-β-d-glucan in other species which is commonly composed of both DP3 and DP4 at different proportions, the (1,3;1,4)-β-d-glucan extracted from brown algae is exclusively built by DP3 as repeating units, as observed by high performance anion-exchange chromatography with pulsed-amperometric detection (HPAEC-PAD) [9]. Other unusual fresh water algae, like *Monodus subterraneus* in Xanthophyceae and dinoflagellate *Peridinium gatunense,* have also been reported to have (1,3;1,4)-β-d-glucans, but the distribution DP3 and DP4 requires further investigation [32,33].

The presence of (1,3;1,4)-β-d-glucan in the cell walls of *Equisetum arvense*, Phaeophyceae (brown algae) in phylum of Stramenopiles that is not closely related to any land plants and green algae, the non-conserved structural characteristics across lineages and the absence of (1,3;1,4)-β-d-glucans in some closely related families of grasses and cereal, suggest synthesis could be highly conserved, or a convergence-independent evolution of (1,3;1,4)-β-d-glucan synthase genes.

#### 2.1.1. Biosynthesis of (1,3;1,4)-β-d-Glucan in Viridiplantae

The *cellulose synthase-like* (*Csl*) gene superfamily is responsible for the biosynthesis of non-cellulosic polysaccharides in plant cell walls, including mannans, glucoxylans, xyloglucans, and (1,3;1,4)-β-d-glucans. These *Csl* genes superfamily encoded transmembrane proteins with up to eight membrane-spanning domains and a highly conserved D,D,D,QxxRW motif in their catalytic sites for substrate binding and catalysis [34]. Within the Csl family, CslF and CslH have been implicated in the biosynthesis of (1,3;1,4)-β-d-glucans in grasses and cereals [35]. To better understand where the biosynthesis of (1,3;1,4)-β-d-glucans takes place, the cellular location of the CslH protein has been investigated, using immunogold-labeled antibodies that target CslH epitopes. In transgenic lines of *Arabidopsis*, CslH proteins were localized in the Golgi vesicles and endoplasmic reticulum (ER), and unexpectedly no labeling was found on the plasma membrane [36]. This finding leads to the assumption that biosynthesis of (1,3;1,4)-β-d-glucans could take place in the Golgi. Biochemical studies of tracking newly synthesized (1,3;1,4)-β-d-glucan incorporated [^14^C]-glucose from UDP-[^14^C] glucose suggested that the synthase activity was found only in Golgi [37]. However, Meikle et al. developed a monoclonal antibody against (1,3;1,4)-β-d-glucan and showed no labelling in the Golgi, but strong labelling of (1,3;1,4)-β-d-glucans at the plasma membrane [38,39]. Due to this discrepancy, a hypothesis called “two-phase process” is proposed. It is hypothesized that the synthesis of (1,3;1,4)-β-d-glucan begins in the Golgi with either CslF or CslH enzymes operating the assembly of oligosaccharides to create smaller building blocks, perhaps (1,4)-linked cellodextrins. These building blocks may not be detectable by the (1,3;1,4)-β-d-glucan-specific antibodies and may be transported from the Golgi by carrier molecules, such as an intermediate cellodextrin-lipid, to the plasma membrane [4]. Subsequently, the smaller building blocks are (1,3)-linked assembled and attached to the cell wall possibly by another glycosyltransferase [2].

Another proposed biosynthetic route is that CslF and CslH could catalyze the two different linkages independently with only one catalytic subunit. Since then, studies have found *CslF6* to be the gene primarily responsible for the biosynthesis of (1,3;1,4)-β-d-glucans as it is highly expressed in many tissues especially in the developing endosperm; additionally, downregulation of (1,3;1,4)-β-d-glucan synthesis was observed when the *CslF6* gene was knockdown, knockout or partially loss-of-function [40,41,42,43,44]. Furthermore, CslF6 expressed into tobacco cells and *Pichia* was reported to be able to synthesize both (1,3)- and (1,4)-β-linkages of (1,3;1,4)-β-d-glucans. The (1,3;1,4)-β-d-glucans produced by CslF6 at the Golgi were subsequently observed to be channeled through the secretory pathway to the plasma membrane [45,46]. A recent study revealed that the fine structure of (1,3;1,4)-β-d-glucan can be altered by changing a single amino acid in the active site of CslF6, altering the position and flexibility of the TED motif [47].

#### 2.1.2. Physiological Function of (1,3;1,4)-β-d-Glucan in Viridiplantae

The (1,3;1,4)-β-d-glucans in plants provide structural roles, such as strength, flexibility and elasticity. They are also important in the transport by providing porosity during active growth, and the exchange of water, nutrients and other small molecules, such as phytohormones between adjacent cells [48]. In Poaceae, (1,3;1,4)-β-d-glucan is associated with the elongating cells differentiating from the meristematic cells, and is largely absent in the meristematic cells and the mature tissues where growth has ceased [49,50,51]. Additionally, in the Poaceae (1,3;1,4)-β-d-glucan, around 30% are β-1,3-glycosidic linkages, and these primarily consist of DP3s and DP4s, with only 10% of the oligosaccharides being larger than DP4s. With no consecutive (1,3)-β-bonds existing, (1,3;1,4)-β-d-glucan is linear with “kinks”, caused by the single (1,3)-β-linkage inserted within β-1,4 oligosaccharides, which makes it more flexible and soluble [52]. One could see it as a cis-double bond in lipids, which are harder to stack due to it being folded. It is possible that the irregularity of the (1,3;1,4)-β-d-glucan “kinks” makes the (1,3;1,4)-β-d-glucans unable to lay neatly on each other. (1,3;1,4)-β-d-Glucan is found in abundance in the aleurone layer and endosperm of various grains (like barely or oats), reaching the highest concentrations at the highest rate of growth in the grains. Interestingly, the action of (1,3;1,4)-β-d-glucanase facilitates cell wall turnover, and (1,3;1,4)-β-d-glucan ceases to exist once the cells have completed the expansions, suggesting this is an important component in cell expansion and the early stages of germination. Furthermore, in a study by Vega-Sánchez et al. in 2012, (1,3;1,4)-β-d-glucan was found in the secondary cell wall of the mature stems of rice plants with *CslF6* highly expressed in the young tissue at a greater rate [44]. In addition, the *CslF6* knockout mutant rice had reduced in height by 1/3 and seed production after pollination, also known as seed set, was halved compared to the wild type [44]. The reduction of seed set was most likely the result of deformed male reproductive tissues. The CslF6 is suggested to play an important role in the growth of the rice plants, but it is uncertain if this is due to a lack of (1,3;1,4)-β-d-glucans or some other possible mechanism that the CslF6 might take part in.

Thus far, very little is known about the function of (1,3;1,4)-β-d-glucans in algae. A recent study suggests that (1,3;1,4)-β-d-glucans in brown algae appeared to bond tightly to the cell walls. Their insolubility in water with consistent conformation suggests the structural functions [9].

### 2.2. Fungi and Lichens

Lichens are a combination of fungi and algae or cyanobacteria living in symbiosis, where fungi are recognized as the mycobiont and different algae or cyanobacteria as the photobiont in lichens. (1,3;1,4)-β-d-Glucans have been found in abundance in Parmeliaceae lichens, such as *Cetraria*, *Evernia*, *Newropogon*, *Parmelia*, *Parmotrema*, *Rimelia* and *Usnea* [53]. These (1,3;1,4)-β-d-glucans isolated from lichen species from family Parmeliaceae are also known as lichenin or lichenan. Specifically, the (1,3;1,4)-β-d-glucans extracted from the mycobiont cell wall of *Cetraria islandica* (Iceland moss) gave a higher ratio of DP3 to DP4 (20.2–24.6:1) compared barley and oats (1,3;1,4)-β-d-glucan (1.8–3.5:1) [54]. However, the ratio of (1,4)- to (1,3)-β-linkages in lichenin varies significantly among the lichens, ranging from 2.3:1 found in *C. islandica*, to 0.3:1 in oak moss *Evernia prunastri* [54]. The high proportion of three-linked Glc*p* in *E. prunastri* glucans raised a question of whether both linkages came from two separate glucans.

Besides the lichens, a non-lichen fungus, *Aspergillus fumigatus*, was found to have (1,3;1,4)-β-d-glucans in an alkali-insoluble fraction from its cell wall, which is suggested to be related to fungal cell wall rigidity [55]. The (1,3;1,4)-β-d-glucan was also detected in the cell wall of *Neurospora crassa* with monoclonal antibody [56]. A high percentage of (1,4)-linked glucose was reported in the glycosyl composition and linkage analysis of fungal cell wall from a filamentous fungus *N. crassa* [57]. It is highly likely that the occurrence of (1,3;1,4)-β-d-glucans is not restricted in the *A. fumigatus* and *N*. *crassa*. How the particular variation in the structures of (1,3;1,4)-β-d-glucans found may be related to their function is largely unknown. Other β-glucans, for example, the (1,3;1,6)-β-d-glucan, is a common β-glucan found in the cell walls of various fungal species, and has shown to possess immunomodulatory activities in reducing SARS-CoV-2-induced cytokine storm in the infected patients [58]. Further β-glucan structural survey of taxa in other lichen and fungal families would be interesting to provide evidence in understanding structure–function relationships between β-glucan structures and their functions, and their potential implications for pharmaceutical research and development.

#### 2.2.1. Biosynthesis of (1,3;1,4)-β-d-Glucan in Fungi and Lichens

In 2015, the first fungal (1,3;1,4)-β-d-glucan synthase, Three-four passes 1 (Tft1), was reported in *A. fumigatus* [59]. Although the protein sequence of Tft1 shows only 30% homology to the plant (1,3;1,4)-β-d-glucan synthases, CslF and CslH, the homology was confined strictly within the CesL (Cellulose synthase-like) domain at the active site of the Csl glycosyltransferases. Additionally, the *A. fumigatus tft1Δ* strain lost the ability to produce (1,3;1,4)-β-d-glucans, suggesting the *Tft1* is an essential gene that could encode the (1,3;1,4)-β-d-glucan synthase. To investigate whether orthologs exist in other fungal organisms, the *Tft1* was blasted against the NCBI. Interestingly, the result showed several fungal organisms to contain close orthologs with 75% to 90% identity. In addition, the (1,3;1,4)-β-d-glucan synthase of *Aspergillus nidulans* was reported. This synthase is encoded by *celA,* which is distantly related to plant *Csl* and orthologous to the *Tft1* [60]. Two candidate GT2 enzymes, CPS-1 and NCU03240, are most likely responsible for the synthesis of (1,3;1,4)-β-d-glucans in another fungus *N*. *crassa*, as both of these genes, *cps-1/ncu00911* and *ncu03240,* are highly expressed in the vegetative hyphae, where (1,3;1,4)-β-d-glucan was found in the cell wall [61]. All studies suggest there could be many other fungal species containing (1,3;1,4)-β-d-glucans that are yet to be identified.

#### 2.2.2. Physiological Function of (1,3;1,4)-β-d-Glucan in Fungi and Lichens

As one of the components constituting the fungal cell wall, lichenin is believed to contribute to cell wall rigidity in *A. fumigatus*. However, even as 10% of all the cell wall glucans, (1,3;1,4)-β-d-glucan still seems to be dispensable for the cell growth and fitness of *A. fumigatus,* as no phenotypic differences were observed compared to the wildtype when (1,3;1,4)-β-d-glucan synthase, *Tft1*, was deleted or two-fold overexpressed in in vitro growth. Meanwhile, (1,3;1,4)-β-d-glucan is suggested to be relevant in spore formation and enhances β-1,3 glucan synthesis of *A. fumigatus*, but it would require further studying to confirm if these functions are affected directly by (1,3;1,4)-β-d-glucans [59]. In contrast, in *A. nidulans,* the deletion of the *CelA* gene could cause a “balloon” phenotype, which indicates a weakened cell wall. In addition to the changed morphology, the *CelA* gene-deleted strain showed decreased sensitivity to the drugs against the cell wall, Congo red and dichlobenil. These observations strongly implicate (1,3;1,4)-β-d-glucan in cell wall-related progression of *A. nidulans.* In another study, an orthologous gene to *CelA* was found in the pathogenic fungus *Drechslera teres*, which may be responsible for the production of (1,3;1,4)-β-d-glucans on the cell wall of *D. teres,* the fungal species known to cause diseases such as net blotch in barleys and have a severe economic impact [62]. Whether (1,3;1,4)-β-d-glucan here plays any role could be up for discussion, as the cell wall of various pathogenic species are often relevant for attachment and pathogenicity. Moreover, in *N. crassa*, lichenins contribute to 25% of the vegetative cell wall mass and are thought to cross-link glycoproteins to the cell wall [58].

### 2.3. Bacteria

The presence of (1,3;1,4)-β-d-glucan in bacteria cell walls has been overlooked for many years until the recent discovery of (1,3;1,4)-β-d-glucans in the walls of Gram-negative bacteria *Sinorhizobium meliloti* [10]. Occurrence of (1,3;1,4)-β-d-glucans in the walls of microorganisms such as bacteria confirms that the (1,3;1,4)-β-d-glucan is not unique to planta and fungi, but further questions remain as to how the bacterial (1,3;1,4)-β-d-glucans structure and biosynthesis differ to the planta and fungal (1,3;1,4)-β-d-glucans.

**Figure 2:**
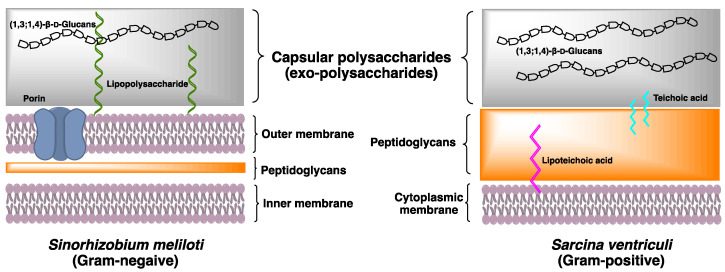
Cell wall architecture of Gram-negative bacteria *Sarcina ventriculi* and Gram-negative bacteria *Sinohizobium meliloti*. Both species have unique capsular polysaccharide (1,3;1,4)-β-d-glucans deposited on the surface of bacteria.

Bacterial cell wall is a complex multilayered structure that protects the bacteria from an unpredictable and often hostile environment. Most bacteria, except mycoplasmas, have a complex cell wall composed of a mixture of polymers made of carbohydrates and amino acids. These are the peptidoglycan polymers that can provide cell rigidity and act as a physical barrier between the cell and its surrounding. In response to the different environment, some species of Gram-positive bacteria and Gram-negative bacteria secrete high concentration of the exopolysaccharides (EXPs) deposited onto the bacterial surface (Figure 2). Their roles include basic functions such as maintaining structural integrity and preventing desiccation, to more complex activities of facilitating the interaction within bacterial communities. The bacterial EXPs have been found in many pathogenic bacteria and have a direct impact on human health because of their ability to form multicellular conglomerates called biofilms.

A recent study reported that the structure of (1,3;1,4)-β-d-glucan in Gram-negative bacteria *S. meliloti* is distinctive to the (1,3;1,4)-β-d-glucans of land plants and fungi, for its entire structure is composed of disaccharide repeating units Glc*p*-β-1,3-Glc*p* (DP2s) [10]. Methylation analysis showed that the partially methylated alditol acetates (PMAAs) derived from (1,3;1,4)-β-d-glucans of *S. meliloti* corresponded to (1,3)- and (1,4)-linked glucopyranosyl residues in a 1:1 ratio. Further structural investigation of (1,3;1,4)-β-d-glucans, using two-dimensional Nuclear Magnetic Resonance with Rotating-frame nuclear Overhauser Effect Spectroscopy (2D-NMR ROESY) spectrum, revealed the cross-peaks between H1 of (1,4)-linked Glc*p* and H3 of (1,3)-linked Glc*p*, and between H1 of (1,3)-linked Glc*p* and H4 of (1,4)-linked Glc*p*, corresponded to a -3)-β-d-Glc*p*-(1,4)-β-d-Glc*p*(1- repetitive polymeric structure. Lichenase digestion only releases DP2, confirming the presence of a novel (1,3;1,4)-β-d-glucan in *S. meliloti*.

Following the discovery of (1,3;1,4)-β-d-glucan in *S. meliloti*, we began to look into bacterial (1,3;1,4)-β-d-glucans that may have been overlooked in early literature. Gram-positive bacteria *Sarcina ventriculi,* a highly robust mesotrophic bacterial species commonly found in the soil and occasionally in human gastrointestinal tracts, can secrete and subsequently deposit an additional thick layer of EXPs onto the bacterial cellular surface [63]. Early X-ray diffraction study showed *Sarcina* EXPs to have similar diffraction pattern to the (1,3;1,4)-β-d-glucans in grasses and cereals. Studies using lichenase digest have confirmed that the (1,3;1,4)-β-d-glucan of *S. ventriculi is* composed of DP3 G4G3G_R_, of which the oligosaccharide profiles are similar to the (1,3;1,4)-β-d-glucan recently isolated from the brown algae *Ectocarpus sp.* [9].

#### 2.3.1. Biosynthesis of (1,3;1,4)-β-d-Glucan in Bacteria

The biosynthesis of bacterial exopolysaccharides is known to share some common characteristics to that in plants. For example, the polysaccharide synthases involved in the bacterial cellulose, alginate and poly-β-d-*N*-acetylglucosamine production have similar structural motifs, such as the transmembrane-spanning domain, a catalytic D,D,D,QxxRW motif, and a PilZ domain [64]. These motifs are essential for substrate binding and to catalyze the biosynthesis of bacterial exopolysaccharides. Specifically, the PilZ domain is known to involve in the binding to the secondary messenger cyclic diguanylate (c-di-GMP). Two putative genes *bgsA* and *bgsB* are proposed to be involved in the biosynthesis of *S. meliloti* (1,3;1,4)-β-d-glucan. In silico study suggests that the BgsA protein has seven-transmembrane spanning domains and a catalytic D,D,D,QxxRW motif, whereas the PilZ domain is absent. Biochemical study of heterologously expressed BgsA *C*-terminal domain consisting of 139 amino acids suggests that this unknown domain is also involved in the binding of c-di-GMP, but the lack of PilZ suggests that the activation mechanism of BgsA for the production of (1,3;1,4)-β-d-glucans could be different to the biosynthesis of other exopolysaccharides. A recent study showed that putative (1,3;1,4)-β-d-glucan synthase, the *bgsA* and *bgsB* operons, have been identified in the genomes of *Rhizobium*, *Agrobacterium* and *Methylobacterium*, all within the order Rhizobiales [10], suggesting that the occurrence of (1,3;1,4)-β-d-glucan in bacteria maybe more frequent than we have previously expected.

#### 2.3.2. Physiological Function of (1,3;1,4)-β-d-Glucan in Bacteria

As (1,3;1,4)-β-d-glucans in bacterial species are quite a novel finding, there are few studies investigating the functional properties of bacterial (1,3;1,4)-β-d-glucans. However, in the Gram-negative bacterium *S. meliloti*, where the (1,3;1,4)-β-d-glucan is found exterior to the outer membrane and consists exclusively of DP2 as repeat units, a conclusion has been made that this water-insoluble (1,3;1,4)-β-d-glucan with high production rate promotes the accessibility of aggregation and biofilm production. As both of these processes occur around plant roots, the study suggests that the *S. meliloti* (1,3;1,4)-β-d-glucan could be important for bacteria adherence to plant surfaces [10].

## 3. Conclusions

(1,3;1,4)-β-d-Glucans are found most commonly in higher plants such as grasses and cereals, and less commonly in the walls of monilophyte *Equisetum*, some bryophytes, algae, lichens, fungus, a chromalveolate, and two bacterial species. Except for a few taxa, structural variation of (1,3;1,4)-β-d-glucans across lineage is particularly apparent, but little is known about their physicochemical properties and their biosynthesis [10]. This information is needed to understand the evolution of the important cell wall polysaccharides, the (1,3;1,4)-β-d-glucans, as well as for the possibility of greater utilization of the glucans in food and beverage, and biotechnology industries.

## Figures and Tables

**Table 1:** Ratio of mixed-linkage glucan (MLG) oligosaccharides produced from different species after lichenase digestion.

Species	DP2	DP3	DP4	References
*Hordeum vulgare*	0	1.8–3.5	1	[6]
*Triticum aestivum*	0	3.0–4.5	1	[11,12]
*Avena sativa*	0	1.5–2.3	1	[6]
*Secale cereale*	0	1.9–3	1	[6]
*Equisetum arvense*	0	0.05–0.1	1	[3]
*Equisetum fluviatile*	0	0.1	1	[7]
*Cetraria islandica*	0	20.2–24.6	1	[6]
*Sinorhizobium meliloti*	1	0	0	[10]
*Sarcina ventriculi*	0	1	0	[13]
*Ectocarpus sp.*	0	1	0	[9]
